# Predictive value of serum HIF-1α/HIF-2α and YKL-40 levels for vascular invasion and prognosis of follicular thyroid cancer

**DOI:** 10.1016/j.clinsp.2024.100486

**Published:** 2024-09-14

**Authors:** Jiulong Li, Kuai Yu, Dingchuan Chen, Guangcheng Luo, Jiedeng Jia

**Affiliations:** a Department of Clinical Laboratory, Gaoping District People's Hospital of Nanchong, Nanchong City, Sichuan Province, China; b Department of Clinical Laboratory, The People's Hospital of Wusheng, Guang'an City, Sichuan Province, China; c Department of Clinical Laboratory, Sichuan Gem Flower Hospital, Chengdu City, Sichuan Province, China; d Department of Clinical Laboratory, Affiliated Hospital of North Sichuan Medical College, Nanchong City, Sichuan Province, China; e Department of Vascular Surgery, Gaoping District People's Hospital of Nanchong, Nanchong City, Sichuan Province, China

**Keywords:** Hypoxia inducible factor-1α, Chitinase 3-like protein 1, Follicular thyroid carcinoma, Vascular Invasion, Recurrence-Free Survival

## Abstract

**Objective:**

This study investigated the significance of serum hypoxia-inducible factor (HIF)-1α/HIF-2 α and Chitinase 3-Like protein 1 (YKL-40) levels in the assessment of vascular invasion and prognostic outcomes in patients with Follicular Thyroid Cancer (FTC).

**Methods:**

This prospective study comprised 83 patients diagnosed with FTC, who were subsequently categorized into a recurrence group (17 cases) and a non-recurrence group (66 cases). The pathological features of tumor vascular invasion were classified. Serum HIF-1α/HIF-2α and YKL-40 were quantified using a dual antibody sandwich enzyme-linked immunosorbent assay, while serum Thyroglobulin (Tg) levels were measured using an electrochemiluminescence immunoassay method. The Spearman test was employed to assess the correlation between serum factors, and the predictive value of diagnostic factors was determined using receiver operating characteristic curve analysis. A Cox proportional hazards regression model was utilized to analyze independent factors influencing prognosis.

**Results:**

Serum HIF-1α, HIF-2α, YKL-40, and Tg were elevated in patients exhibiting higher vascular invasion. A significant positive correlation was observed between Tg and HIF-1α, as well as between HIF-1α and YKL-40. The cut-off values for HIF-1α and YKL-40 in predicting recurrence were 48.25 pg/mL and 60.15 ng/mL, respectively. Patients exceeding these cut-off values experienced a lower recurrence-free survival rate. Furthermore, serum levels surpassing the cut-off value, in conjunction with vascular invasion (v2+), were identified as independent risk factors for recurrence in patients with FTC.

**Conclusion:**

Serum HIF-1α/HIF-2α and YKL-40 levels correlate with vascular invasion in FTC, and the combination of HIF-1α and YKL-40 predicts recurrence in patients with FTC.

## Highlights

• Clinicopathologic characteristics of patients.
• Relationship between the degree of VI and serum HIF-1α, HIF-2α, YKL-40 and off-sTG levels.
• Correlation analysis of serum HIF-1α, HIF-2α, and sYKL-40 with disease metastasis and recurrence in FTC patients.
• Predictive assessment of serum HIF-1α, HIF-2α, and YKL-40 levels on the degree of VI and prognosis of FTC patients.

## Introduction

The incidence of thyroid cancer continues to rise worldwide, making it one of the most common endocrine cancers.^1^ In thyroid cancer, Follicular Thyroid Carcinoma (FTC) represents approximately 10 %‒15 % of malignant tumors.^2^ Clinically, FTC is generally diagnosed by histopathologic findings of peritumoral infiltration and/or Vascular Invasion (VInv) and classified as minimally invasive, encapsulated angioinvasive, and widely invasive.^3^ FTC metastasizes more frequently to distant organs via the bloodstream.^4^ Cancer that infiltrates peripheral structures can lead to local recurrences, distant metastases, and higher mortality rates.^5^ It is well established that VInv plays a significant role in the prognosis of patients with tumors.^6^, ^7^, ^8^ Notably, a higher degree of VInv has been reported to be associated with a higher recurrence rate as well as poorer recurrence-free survival.^9^ However, clinically, the degree of VInv is mainly visualized by microscopic observation of pathological specimens. Therefore, noninvasive markers are urgently needed to accurately analyze the degree of VInv and to assess the prognosis of FTC patients.

Tumorigenesis and progression are largely dependent on tumor vasculature.^10^^,^^11^ A large number of pro- and anti-angiogenic molecules have been identified.^12^^,^^13^ The inevitable hypoxic microenvironment in solid tumors is a major contributor to tumor angiogenesis.^14^ Hypoxia-Inducible Factors (HIF) are the major transcriptional regulators of cellular responses to hypoxia.^15^ Two of the major isoforms, 1α and 2α, regulate many potent pro-angiogenic and anti-angiogenic molecules.^16^ It has been demonstrated that HIF-1α and HIF-2α are highly expressed in tumors with VInv^17^ and are involved in FTC.^18^ However, the association of HIF-1α and HIF-2α with the degree of VInv and prognosis of FTC patients is unclear.

Chitinase 3-Like protein 1 (YKL-40), a secreted glycoprotein, is expressed at elevated levels in a variety of advanced human cancers.^19^ YKL-40 induces tumor microangiogenesis, cell survival, proliferation, tissue remodeling, and immunomodulation.^20^ Serum YKL-40 can be identified as a prognostic indicator for endometrial cancer patients.^21^ In addition, YKL-40 is highly expressed in cancer tissues in patients with renal cancer.^22^ Currently, no studies have reported the value of YKL-40 in predicting the degree of VInv and prognosis in patients with FTC. Thyroglobulin (Tg), especially stimulated Tg (sTg), is highly sensitive in predicting the postoperative status of patients with thyroid cancer, including recurrence^23^ and death.^24^

To date, several clinical studies have independently investigated the correlation between clinicopathologic features, degree of VInv, and prognosis in patients with FTC. To our best knowledge, no clinical studies have investigated the levels of serum HIF-1α, HIF-2α, and YKL-40 in FTC patients with different degrees of Vinv. In this study, serum HIF-1, HIF-2, and YKL-40 were examined for their relationship to Vinv in patients with FTC and their predictive value about the prognosis of these patients.

## Materials and methods

### Patients

This study was a prospective study, and the standard for reporting prognostic studies follows (STARD). We recruited 89 patients with pathological diagnosis of FTC who underwent surgery at Gaoping District People's Hospital of Nanchong during the period 2017.01–2022.06. Follow-up was cut off in November 2023, with a minimum follow-up period of 12 months and a maximum follow-up period of 69 months. All Hematoxylin-Eosin (HE)-stained sections of these cases were reviewed by an expert pathologist.

Inclusion criteria: (1) Age greater than 18 years old; (2) Undergoing surgery in the hospital and pathological diagnosis of FTC; (3) Complete clinical counts and follow-up information. Regular postoperative visits were made through follow-up visits, telephone and internet contact, every 3 months for the first 3 years after surgery and every 6 months after 3 years.

Exclusion criteria: (1) Patients diagnosed with poorly differentiated carcinoma, patients with other coexisting thyroid malignancies; (2) Patients with insufficient postoperative follow-up period (< 12-months); (3) Patients diagnosed with distant metastases prior to surgery; (4) Patients with a family history of thyroid cancer.

The study was approved by the Ethics Committee of Gaoping District People's Hospital of Nanchong (n° 201612NC15). All subjects submitted an informed consent form at enrollment.

### Sampling and laboratory testing

The quality control serum was a multi-level constant-value quality control serum from BIO-RAD (USA). Fasting venous blood was sampled before operation or review, and the serum was separated within 30 min and tested directly or stored in liquid nitrogen. Serum TSH and Tg were measured by electrochemiluminescence immunoassay. The normal range of TSH was 0.4∼4.2 mU/L, and the detection range was 0.004∼140.00 mmoL/L. The detection range of Tg was 0.04∼1000.00 μg/L, and TG is defined as the value of Tg after discontinuation of euthyrox or stimulation of TSH for 4-weeks (TSH > 30 mU/L). Tg > 2 ng/mL may be a highly sensitive indicator of the presence of cancer cells. Serum HIF-1α, HIF-2α, and YKL-40 were measured by Enzyme-Linked Immunoabsorbant Assay (ELISA) kits from Shanghai yuan Mu Biotechnology Co., Ltd. and R&D Systems Europe Ltd, respectively. The standard product and HRP-labeled detection antibodies were sequentially added to the microtiter wells captured in the embedded microtiter wells. After propagation and thorough washing, the color development reaction was performed using tetramethylbenzidine. Color intensity was measured using a photometer at 450 nm and 490 nm. Concentrations were calculated based on the concentration of standard samples and the spectrophotometric value of each well.

### Histopathologic examination of VInv

H&E-stained tumor sections of each patient were assessed by 2 pathologists. VInv grading was assessed based on the number and maximum size of the involved vessels. To assess the number of VInv, the total number of VInv in all sections examined was divided by the number of sections observed on each slide. The degree of VInv was categorized as: v0, no VInv; v1+, minimal VInv (one or two vascular invasive foci); and v2+, moderate VInv (three or four foci).^24^

### Data statistics

Categorical variables were expressed as frequencies (%). Continuous variable distributions were assessed using the Kolmogorov-Smirnov test and expressed as median [quartiles]. Mann-Whitney *U* or Kruskal-Wallis tests were performed for comparisons between two or more independent samples. Correlations between variables were calculated using nonparametric Spearman's method. Benjamini-Hochberg False Discovery Rate (FDR) analysis was performed to calculate the corrected p-value, and adjusted *p* < 0.05 was statistically significant. The prognostic endpoint was that the patients had recurrence. By drawing the Receiver Operating Characteristic (ROC) curve, the Area Under the Curve (AUC) was calculated, and the Youden cut-off value was obtained to determine the predictive value of preoperative HIF-1α, HIF-2α, and YKL-40 levels for VInv and prognosis. Factors affecting prognosis were screened using Cox regression modeling. Recurrence-free survival curves were plotted using the Kaplan-Meier method and analyzed by the log-rank test. Subgroups were generated according to cut-off values, and to investigate the prognostic value of HIF-1 versus YKL-40, univariate and multivariate Cox proportional risk models were used. Statistical significance was assessed using a two-tailed test, with p-value < 0.05 considered to indicate statistical significance. Statistical tests were performed using IBM SPSS version 22.0, and graphics were produced using GraphPad Prism 9.0.

## Results

### Clinicopathologic characteristics of patients

In the present study, 89 patients with FTC were followed, of whom 4 developed metastatic disease and 2 died. The clinicopathological characteristics of the remaining 83 patients are summarized in Table 1. Of the 83 patients, the maximum follow-up period was 70 months, and 17 patients (20.5 %) relapsed during the follow-up period. The age range of patients was 24.3‒68.6 years. According to the postoperative pathological diagnosis, 46 cases (69.7 %) were minimally invasive, 26 cases (31.3 %) were encapsulated angioinvasive, and 11 cases (13.2 %) were widely invasive. Ten cases diagnosed as widely invasive during the follow-up time experienced recurrence. A total of 11 patients (13.2 %) underwent total thyroidectomy as a first surgical intervention. Of the 62 patients who did not undergo total thyroidectomy, 5 patients (6.0 %) underwent total thyroidectomy as a secondary surgical intervention based on the physician's recommendation (mainly due to extensive tumor infiltration). Twenty-four patients (28.9 %) received radioiodine administration in varying doses (13‒100 mCi) after thyroidectomy. The authors observed a correlation between age ≥ 55 years, tumor > 4 cm, pathological diagnosis of widely invasive, and adjuvant therapy postoperatively with recurrence in patients with FTC.

**Table 1:** Clinical baseline characteristics of relapsed and non-relapsed patients.

**Variable**	**Non-recurrence (*n* = 66)**	**Recurrence (*n* = 17)**	**p-value**
**Age (years)**			0.002
≥ 55	17 (25.8 %)	11 (64.7 %)	
< 55	49 (74.2 %)	6 (35.3 %)	
**Gender**			0.647
Male	39 (59.1 %)	9 (52.9 %)	
Female	27 (40.9)	8 (47.1)	
**Tumor size, (cm)**			0.002
> 4	14 (21.2 %)	10 (58.8 %)	
≤ 4	46 (78.8 %)	7 (41.2)	
**Histologic subtype**			0.000
Minimally invasive	43 (65.2 %)	3 (17.6 %)	
Encapsulated angioinvasive	20 (30.3 %)	6 (35.3 %)	
Widely invasive	3 (4.5 %)	8 (47.0 %)	
**Extent of thyroidectomy at initial surgery**			0.043
Total	6 (9.1 %)	5 (29.4 %)	
Non-total	60 (90.9 %)	12 (70.6 %)	
**Postoperative adjuvant therapy**			0.014
No	51 (77.3 %)	8 (47.1 %)	
Radioiodine therapy	15 (22.7 %)	9 (52.9 %)	
**TSH (μIU/mL, M[IQR])**			0.473
Normal	52 (78.8 %)	12 (70.6 %)	
Down or up	14 (21.2 %)	5 (29.4 %)	

Data are shown as n (%) and differences between groups were determined using the Pearson Chi-Square test; p-value < 0.05 was statistically significant.

### Relationship between the degree of VInv and serum HIF-1α, HIF-2α, YKL-40 and off-sTG levels

**Fig. 1:**
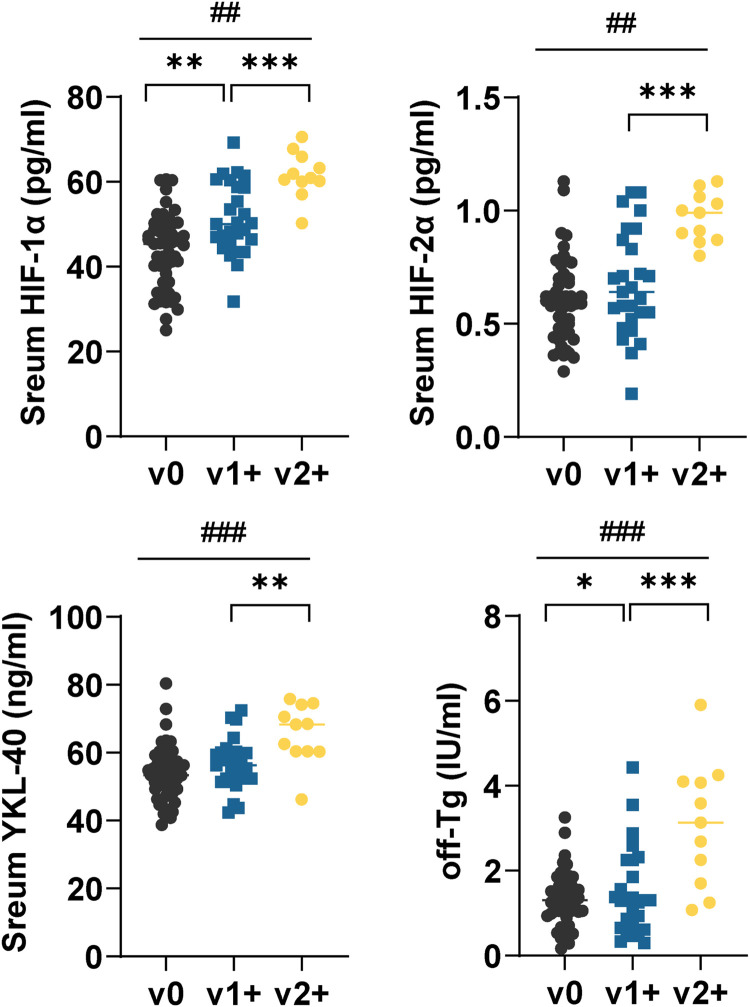
**Serum HIF-1α, HIF-2α, YKL-40, and off-sTG in FTC patients with different degrees of VInv.** Each point represents a case. Mann-Whitney *U* test or Kruskal-Wallis was used to compare differences between groups. off-sTG was defined as the last sTG before the termination of follow-up, i.e., for patients with recurrence it was the last sTG before recurrence. Comparisons between the two groups *p < 0.01, **p < 0.001, and comparisons between the three groups ##*p* < 0.01, ####*p* < 0.001.

Serum HIF-1α, HIF-2α, YKL-40, and off-sTG were higher in v2+ patients than in v1+ patients (*p* < 0.01) (Fig. 1). Serum HIF-1α and off-sTG levels were correlated with the degree of VInv, and serum levels of these two indicators increased with the degree of VInv (Fig. 1).

### Correlation analysis of serum HIF-1α, HIF-2α, and YKL-40 with disease metastasis and recurrence in FTC patients

**Fig. 2:**
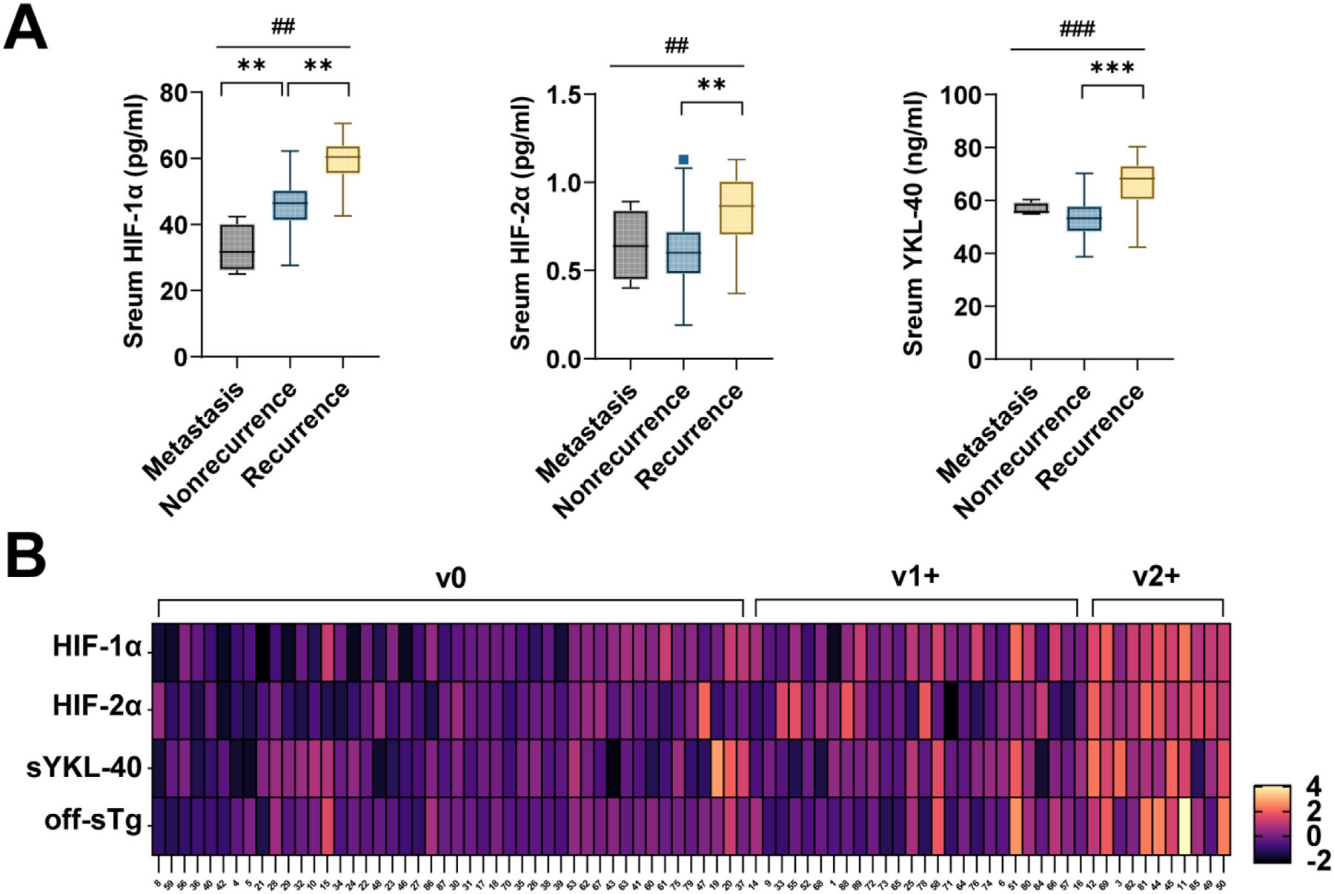
**Correlation analysis of serum HIF-1α, HIF-2α, and YKL-40 with disease metastasis and recurrence in FTC patients.**(A) Heat map analysis of serum HIF-1α, HIF-2α, and YKL-40 with the degree of VInv using Z-Score standardized data. (B) Levels of serum HIF-1α, HIF-2α, and YKL-40 in patients with metastatic and recurrent cancer. Data are expressed as M [IQR] and Mann-Whitney *U* test or Kruskal-Wallis were used to compare differences between groups (*p < 0.01, **p < 0.001 for comparisons between two groups and #*p* < 0.001 for comparisons between three groups).

After excluding 2 cases of death, serum HIF-1α, HIF-2α, and YKL-40 had higher levels in recurrent patients relative to metastatic and non-recurrent patients (*p* < 0.01) (Fig. 2A). In addition, the authors did not find significant differences between serum HIF-2α and YKL-40 in metastatic and non-recurrent patients. Further, serum HIF-1α, HIF-2α, and YKL-40 were analyzed by correlation heatmap with the degree of VInv in recurrent and non-recurrent patients. The results showed that v2+ patients were well stratified with v1+ and v0 (Fig. 2B). Subsequently, by nonparametric spearman correlation analysis, as shown in Table 2, Tg, last measured before the follow-up cutoff, had a significant positive correlation with HIF-1α (*r* = 0.592, *p* = 0.000) and YKL-40 (*r* = 0.449, *p* = 0.000). Notably, there was also a significant positive correlation between HIF-1α and YKL-40 (*r* = 0.398, *p* = 0.000). However, the authors did not find statistically significant correlations between HIF-2α and other biomarkers.

**Table 2:** Correlation analysis between serum HIF-1α, HIF-2α, YKL-40 and off-TG in FTC patients.

**Indices**	**HIF-1α**	**HIF-2α**	**YKL-40**	**off-Tg**
HIF-1α	*r* = 1			
HIF-2α	*r* = 0.229	*r* = 1		
	*p* = 0.00			
YKL-40	*r* = 0.398	*r* = 0.142	*r* = 1	
	*p* = 0.000	*p* = 0.201		
off-Tg	*r* = 0.592	*r* = 0.203	*r* = 0.449	*r* = 1
	*p* = 0.000	*p* = 0.066	*p* = 0.000	

The data in the table is shown as *r* (correlation coefficient), 0.1‒0.3: Weak correlation; 0.3‒0.5: Moderately strong correlation; 0.5‒1.0: Strong correlation. Non-parametric spearman correlation analysis followed by FDR correction was used and p-value < 0.05 was statistically significant.

### Predictive assessment of serum HIF-1α, HIF-2α, and YKL-40 levels on the degree of VInv and prognosis of FTC patients

**Fig. 3:**
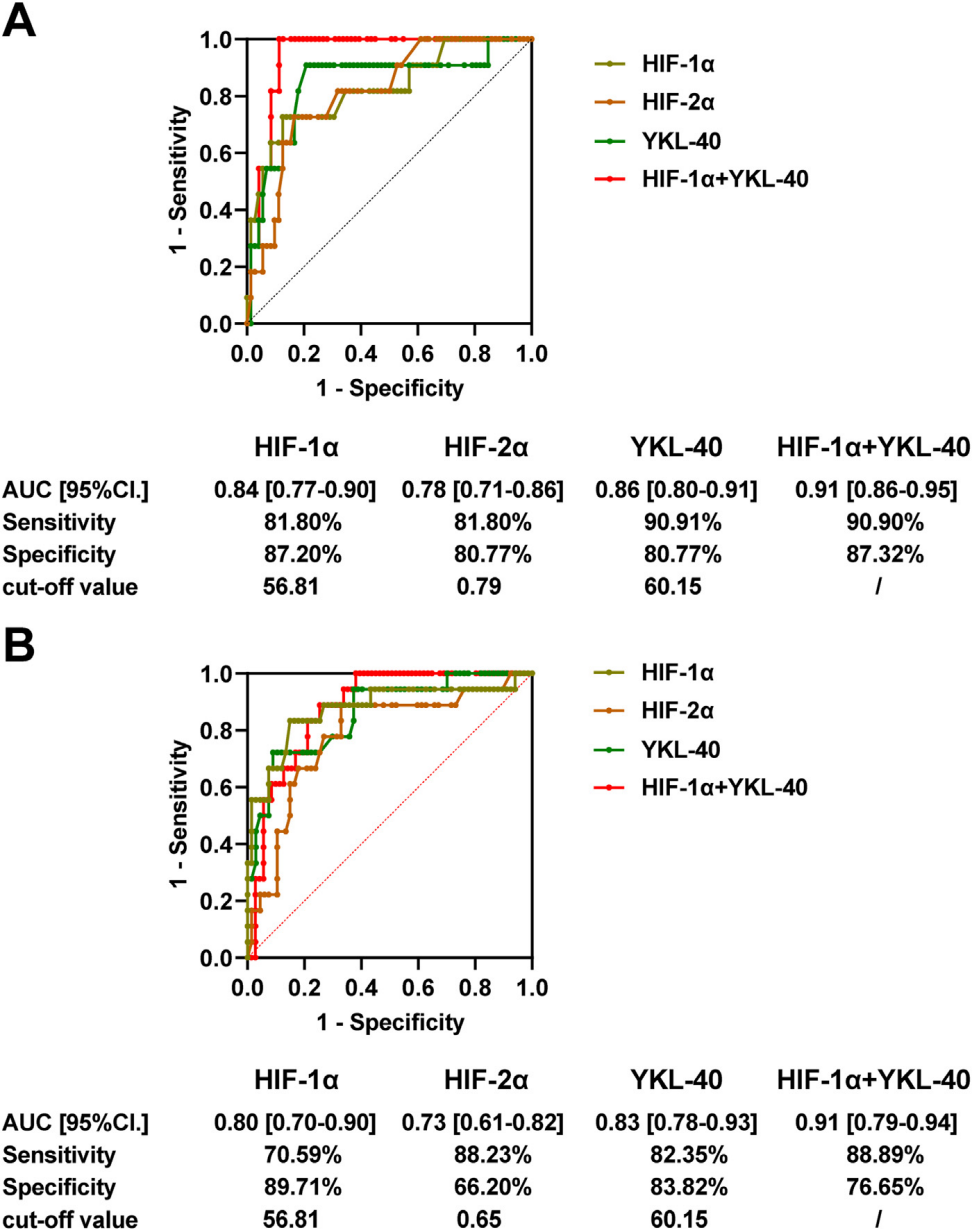
**Assessment of the predictive potency of serum HIF-1α, HIF-2α, and YKL-40 levels on the degree of VInv and recurrent FTC patients.**(A) VInv and (B) disease recurrence was assessed by ROC curves and AUC. Serum HIF-1α and YKL-40 levels were used as covariates to obtain predictive probabilities by binary logistic, and the predictive probabilities were subsequently included in the test variables to obtain the ROC curve analysis. The maximum Youden index was calculated based on the AUC; *p* < 0.05 is statistically significant.

The predictive value of the above indicators on the degree of VInv and recurrence in FTC patients was analyzed by ROC curve and AUC. Serum HIF-1α, HIF-2α, and YKL-40 levels had high diagnostic values for the degree of VInv (Fig. 3A) and postoperative recurrence (Fig. 3B) in FTC patients. The cut-off values of serum HIF-1α, HIF-2α, and YKL-40 levels to distinguish the degree of v0/v1+ from v2+ VInv were 56.81 pg/mL, 0.79 pg/mL, and 60.15 ng/mL, respectively. Notably, the cutoff values of serum HIF-1α versus YKL-40 levels in distinguishing recurrence in FTC patients were consistent with those in distinguishing the degree of VInv (Fig. 3A‒B). Based on the spearman correlation analysis, there was a significant correlation between HIF-1α and YKL-40. The combination of HIF-1α and YKL-40 was further analyzed, and as shown in Fig. 3, the AUC values of the combined metrics were higher than those of independent detection of HIF-1α or YKL-40 in assessing the degree of VInv and recurrence.

**Fig. 4:**
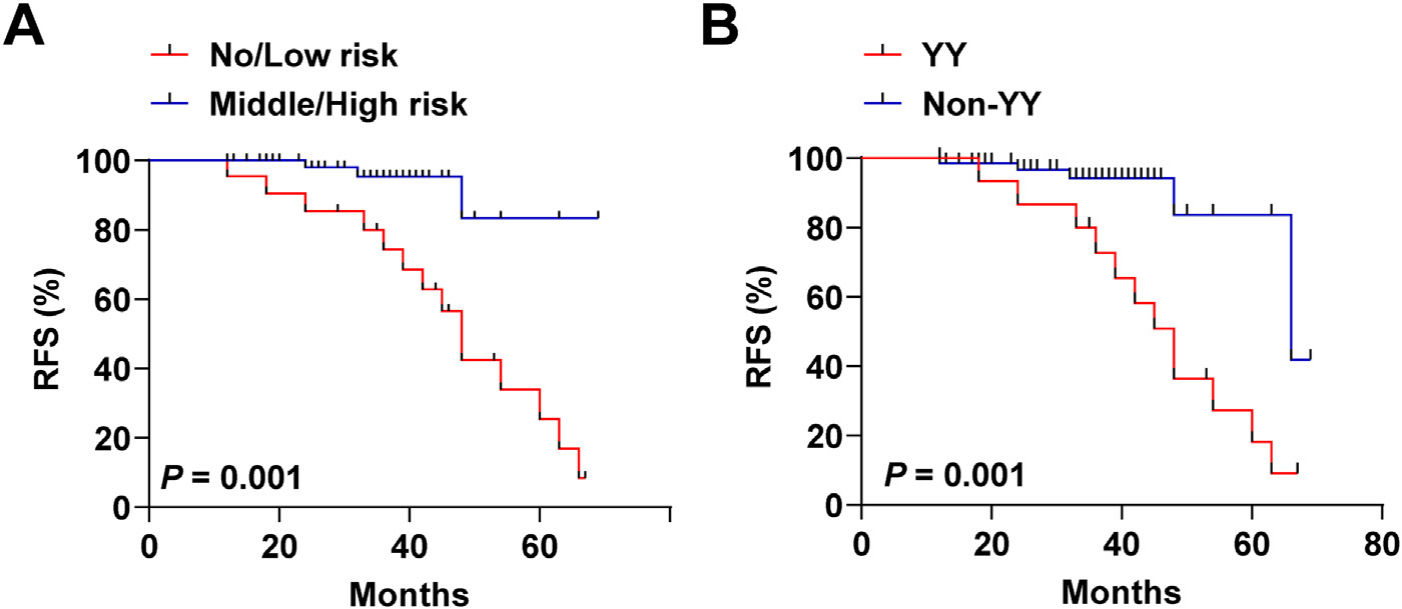
**Recurrence-free survival curves of FTC patients subgrouped based on cutoff values.** Stratification based on the cutoff values of HIF-1α (56.81 pg/mL), HIF-2α (0.65 pg/mL), and YKL-40 (60.15 ng/mL) in predicting the degree of VInv was performed. (A) Recurrence-free survival curves of patients with no/low-risk versus intermediate/high-risk FTC. Stratification based on cut-off values of HIF-1α (48.25 pg/mL) and YKL-40 (60.15 ng/mL) in predicting recurrence in patients with FTC, YY: serum HIF-1α > 48.25 and YKL-40 > 60.15; Non-YY: serum HIF-1α ≤48.25 or YKL-40 ≤ 60.15. (B) Recurrence-free survival curves for YY versus Non-YY patients. Kaplan-Meier curves and two-tailed log-rank tests were used to describe patient recurrence survival, ***p < 0.001; **p < 0.01; *p < 0.05. Points on the curves represent lost events or recurrent events.

The authors categorized patients into no/low-risk and intermediate/high-risk categories by distinguishing cutoff values for the degree of VInv. As shown in Fig. 4A, intermediate/high risk patients had a poorer recurrence-free survival curve, i.e., lower RFS, relative to no/low-risk FTC patients (*p* = 0.001). Subsequently, the authors categorized the patients into 2 categories to differentiate patients from recurrence or non-recurrence (HIF-1α, 48.25 pg/mL; YKL-40, 60.15 ng/mlL). A total of 16 patients were enrolled in the YY subgroup. Twelve of these patients relapsed during follow-up. As shown in Fig. 4B, patients in the YY subgroup also had poorer RFS relative to the Non-YY group. Finally, to identify predictors of prognosis in patients with FTC, multivariate Cox proportional risk analyses were performed, and variables showing significance in univariate analysis were used as covariates. Clinical characteristics, including age, gender, tumor size, extent of initial surgical thyroidectomy, postoperative adjuvant therapy, and degree of VInv were used as adjusting factors for Cox analysis. As shown in Table 3, v2+ was (HR = 1.13, 95 % CI 1.04‒1.75, *p* = 0.035) an independent factor affecting recurrence in patients with FTC. In addition, tumor size > 4 cm (HR = 1.10, 95 % CI 1.00‒1.32, *p* = 0.012) and HIF-1α with YKL-40 serum level above the cut-off value (HR=1.49, 95 % CI 1.30‒1.91, *p* = 0.001) were also independent prognostic factors for FTC.

**Table 3:** Univariate and multivariate Cox proportional risk regression analysis.

**Variable**	**Univariate**			**Multivariate**		
	**OR**	**95 % CI**	**p-value**	**OR**	**95 % CI**	**p-value**
**Age (years)**						
≥ 55						
< 55	1.33	0.56‒2.48	0.556			
**Gender**						
Male						
Female	2.05	0.48‒5.12	0.412			
**Tumor size (cm)**						
≤ 4						
> 4	1.72	1.31‒2.25	0.001	1.10	1.00‒1.32	0.012
**Extent of thyroidectomy at initial surgery**						
Total						
Non-total	0.68	0.44‒4.68	0.748			
**Postoperative adjuvant therapy**						
No						
Radioiodine therapy	1.08	0.67‒4.33	0.265			
**VInv**						
v0/v1+						
v2+	1.92	1.44‒2.42	0.000	1.13	1.04‒1.75	0.035
**HIF-1α >48.25 and YKL-40 >60.15**						
No						
Yes	2.05	1.56‒2.76	0.001	1.49	1.30‒1.91	0.001

## Discussion

Angiogenesis occurs abnormally in tumors during which new blood vessels are generated and germinated at the tumor site. These successive steps are critical for tumor progression.^25^ The prognosis of many cancer patients also depends on VInv. VInv has been demonstrated to be a valuable potential biomarker to predict distant metastasis and cancer recurrence.^26^ Identifying biomarkers linked to tumor progression can aid in evaluating risks before surgery, detecting tumors, and directing treatment plans. In the present study, preoperative serum HIF-1α, HIF-2α, and YKL-40 levels were higher in patients with recurrent FTC than in patients without recurrence and their levels increased with the degree of VInv. To our knowledge, this is the first study to demonstrate that the degree of VInv correlates with serum HIF-1α, HIF-2α, and YKL-40 levels in patients with FTC. VInv of PTC tumors is found to be associated with a higher rate of tumor recurrence in a retrospective analysis of clinical data from 536 PTC patients.^27^ In the present study, the authors evaluated the value of serum HIF-1α, HIF-2α, and YKL-40 levels in predicting the degree of VInv in patients with FTC and analyzed the predictive value of these indices in the prognosis of patients with FTC.

The concept of Risk of Malignancy (ROM) plays a crucial role in the diagnosis and management of thyroid diseases, specifically in evaluating the probability of malignancy in thyroid nodules. The 2017 Bethesda Thyroid Cell Pathology Reporting System (TBSRTC) and its subsequent versions have suggested varying ROM ranges for follicular tumors or suspected follicular tumors, with the latest 2023 version proposing a more specific average ROM risk of 30 % for such tumors.^28^ The potential for deterioration of follicular tumors is associated with their ability to adapt to hypoxic conditions and invade blood vessels.^29^ In this research, samples were collected through fine needle aspiration of thyroid nodules from patients meeting the criteria outlined in the 2017 Thyroid Bethesda System for Reporting Thyroid Cytopathology.^30^ HIF-1α and HIF-2α in tumors achieve hypoxic adaptation of tumor cells by inducing tumor angiogenesis and altering cellular energy metabolism.^31^ HIF-1α and HIF-2α have been reported to be significantly higher in PTC patients' cancerous tissues.^32^ Pinato D J et al., in 100 cases, found higher levels of HIF-1α expression in patients with advanced tumor progression.^17^ Numerous clinical studies have shown that circulating YKL-40 is elevated in patients with different types of tumors.^33^^,^^34^ Similarly, the present study showed that serum HIF-1α, HIF-2α, and YKL-40 were lower in patients with v0 or v1+. Although VInv is considered to be associated with distant metastasis and recurrence, the authors only observed higher levels of these serum factors in patients with recurrence and lower levels in patients with metastases. The authors believe that this phenomenon may be mainly attributed to the smaller number of metastatic patient cases. In this study, patients were followed up from 12 to 69 months, with a median follow-up of 36 months, using the time a patient underwent surgery as the starting point and recurrence as the prognostic endpoint. In the follow-up study, the authors excluded patients who developed tumor metastasis and died during follow-up (6 cases in total). Despite the favorable long-term prognosis of patients with differentiated thyroid cancer (both PTC and FTC), 15 %‒30 % still recur within 5‒10 years.^35^ During follow-up, cancer recurrence occurred in 20.48 % (17/83) of patients. Of the 17 recurrent patients, 8 of them had moderate VInv (v2+).

Tg is a useful biomarker for predicting tumor remnants or recurrence after surgery in patients with differentiated thyroid cancer.^36^ To better assess the value of these serum biomarkers in recurrence, the authors analyzed the relationship between patients' Tg levels and serum HIF-1α, HIF-2α and YKL-40 levels during recurrence. The results showed that there was a significant correlation between Tg and HIF-1α and YKL-40 and a significant correlation between HIF-1α and YKL-40. HIF-1α and HIF-2α are a pair of structurally similar factors, but their mechanisms in regulating tumor angiogenesis are not identical.^37^ HIF-1 and YKL-40 are related to inflammatory responses in macrophages in the tumor microenvironment.^38^ The findings of this study led us to hypothesize that HIF-1 and YKL-40 promote tumorigenesis and progression synergistically in patients with FTC.

To further evaluate the value of serum HIF-1α, HIF-2α, and YKL-40 in predicting VInv and recurrence, the authors performed ROC analysis and obtained serum HIF-1α, HIF-2α, and YKL-40 cutoff values for predicting VInv (v2+) and recurrence. Consistent with expectations, these biomarkers had high predictive value in differentiating the degree of VInv from recurrence. Using the cutoff value for grouping, the intermediate/high-risk group had higher HIF-1α and YKL-40 levels (diagnosis of recurrence or not) and poor recurrence-free survival rates. The larger the preoperative tumor diameter and the lower the degree of tissue differentiation in thyroid cancer patients, the higher the recurrence rate.^39^ Additionally, the authors confirmed that tumor diameter greater than 4 cm and v2+ in FTC patients were independent risk factors for cancer recurrence, with 1.1- and 1.13-times higher risks, respectively. In addition, both HIF-1α and YKL-40 serum levels above the cutoff value were independent risk factors for cancer recurrence in FTC patients.

However, most studies have confirmed the close correlation between VInv and tumor metastasis.^40^ In this study, due to the limited sample size, the authors did not observe a correlation between these serum biomarkers and tumor metastasis. In addition, although this study excluded subjects with <12 months of follow-up, the follow-up period should be extended to further confirm the present findings due to the low recurrence rate of the disease. In conclusion, changes in serum factor levels should be dynamic during the recurrence process.

## Conclusion

Preoperative serum HIF-1α, HIF-2α and YKL-40, especially the combination of HIF-1α and YKL-40, have a predictive value for the degree of VInv and cancer recurrence in FTC patients. In addition, tumor size, degree of VInv, and combined indices above the cut-off level are independent risk factors for predicting recurrence in FTC patients. Serum HIF-1α in combination with YKL-40 is a potentially valuable biomarker for predicting the degree of VInv and prognosis of patients.

## Authors’ contributions

Jiulong Li designed the research study. Kuai Yu and Dingchuan Chen performed the research. Guangcheng Luo and Jiedeng Jia provided help and advice on the experiments. Kuai Yu and Dingchuan Chen analyzed the data. Jiulong Li wrote the manuscript. Jiulong Li and Jiedeng Jia reviewed and edited the manuscript. All authors contributed to editorial changes in the manuscript. All authors read and approved the final manuscript.

## Declaration of competing interest

The authors declare no conflicts of interest.
